# Health and social support services in older adults recently discharged from hospital: service utilisation and costs and exploration of the impact of a home-exercise intervention

**DOI:** 10.1186/s12877-016-0254-x

**Published:** 2016-04-18

**Authors:** I. Farag, K. Howard, S. O’Rourke, M. L. Ferreira, S. R. Lord, J. C. T. Close, C. Vogler, C. M. Dean, R. G. Cumming, C. Sherrington

**Affiliations:** The George Institute for Global Health, Musculoskeletal Division, Sydney Medical School, The University of Sydney, Level 13, 321 Kent Street, Sydney, NSW 2000 Australia; Institute for Choice, University of South Australia, North Sydney, NSW 2060 Australia; Sydney School of Public Health, University of Sydney, Sydney, NSW 2006 Australia; Neuroscience Research Australia, University of New South Wales, Randwick, NSW 2031 Australia; Sydney Medical School, University of Sydney, Sydney, NSW 2006 Australia; Faculty of Human Sciences, Macquarie University, North Ryde, NSW 2113 Australia; Prince of Wales Clinical School, University of New South Wales, Randwick, NSW 2031 Australia

**Keywords:** Hospitalisation, Older people, Resource use, Costs, Mobility improvement

## Abstract

**Background:**

Admission to hospital can lead to persistent deterioration in physical functioning, particularly for the more vulnerable older population. As a result of this physical deterioration, older people who have been recently discharged from hospital may be particularly high users of health and social support services. Quantify usage and costs of services in older adults after hospitalisation and explore the impact of a home-exercise intervention on service usage.

**Method:**

The present study was a secondary analysis of data from a randomised controlled trial (ACTRN12607000563460). The trial involved 340 participants aged 60 years and over with recent hospitalisation. Service use and costs were compared between intervention (12 months of home-exercise prescribed in 10 visits from a physiotherapist) and control groups.

**Results:**

33 % of participants were re-admitted to hospital, 100 % consulted a General Medical Practitioner and 63 % used social services. 56 % of costs were associated with hospital admission and 22 % with social services. There was reduction in General Medical Practitioner services provided in the home in the intervention group (IRR 0.23, CI 0.1 to 0.545, *p* < 0.01) but no significant between-group difference in service use or in costs for other service categories.

**Conclusion:**

There appears to be substantial hospital and social service use and costs in this population of older people. No significant impact of a home-based exercise program was evident on service use or costs.

**Trial registration:**

Australian and New Zealand Clinical Trial Registry ACTRN12607000563460>TrialSearch.

**Electronic supplementary material:**

The online version of this article (doi:10.1186/s12877-016-0254-x) contains supplementary material, which is available to authorized users.

## Background

It is projected that by the year 2050 almost one quarter of the world’s population will be aged 60 years or over, double the current proportion [1]. The economic implications of an ageing population can be attributed mainly to a rise in health-care costs [2]. Increasing age is also associated with an increase in resource utilisation particularly, medical, allied health and social support services [3].

Hospital admissions for older people are increasing with a significant and disproportionate number of days spent in hospital in comparison to the general population [4, 5]. Admission to hospital often results in reduced physical activity levels and has been shown to be associated with a persistent deterioration in physical functioning, particularly for the more vulnerable older population [6, 7]. Fall rates are also increased in the months after discharge from hospital [8].

As a result of the impact of hospitalisation on physical functioning older people who have recently been discharged from hospital may be particularly high users of health and social support services, but this is yet to be well investigated. The availability of accurate information about service use in people who have been in hospital would be valuable in the clinician’s assessment of patient needs, in service planning and economic modelling. The recent focus on early discharge from hospital [9] may have shifted costs from the acute setting to the community [10]; thus emphasizing the increasing importance of consideration of social support costs in planning and modelling.

Exercise interventions have the potential to enhance muscle strength [11], improve balance [12] and prevent falls in community dwelling older people [13]. Several trials evaluating exercise programs have now been conducted in older people who have been in hospital and these indicate that physical functioning can be improved with well-designed exercise intervention programs [12]. The question remains however as to whether, the observed persistent deterioration in physical functioning following hospital admission is associated with increased resource use and whether exercise programs aimed at improving physical functioning can impact on resource use.

This study aimed to quantify the utilisation and associated costs of residential care, hospital, medical, allied and social support services in older people following hospital discharge. In addition, the influence of a home-based exercise intervention on service usage was explored.

## Methods

The present study was a secondary analysis of data from a randomised controlled trial of a home-based exercise program [14]. The protocol for this prospective, assessor-blinded, randomised controlled trial was approved by The University of Sydney Human Research Ethics Committee and written consent was obtained from all participants. The trial was registered prospectively with the Australian and New Zealand Clinical Trials Registry (ACTRN12607000563460) and complied with the Helsinki Declaration on research involving human subjects. The trial involved 340 participants who were discharged from public hospital wards in Sydney Australia with 169 participants randomly allocated to the control group and 171 to the intervention group. Participants were aged 60 years and over who did not have a medical condition that precluded exercise [15]. Other exclusion criteria were: insufficient English language skills to undertake study assessments and interventions, inability to walk one metre with assistance or a walking aid, a diagnosed degenerative neurological disorder, residence in a high-care facility and cognitive impairment (defined as a Folstein Mini-Mental State Examination Score [16] of less than 24 after any acute confusional state has resolved, a commonly accepted cut-off [17]). The baseline assessment was conducted in participants’ homes after the completion of any organised post-hospital rehabilitation and prior to randomisation. Randomisation was performed centrally by an investigator not involved in recruitment or assessment. Stratification variables included study site and falls history in the year prior to study recruitment.

The trial’s primary outcome measures were falls (self-reported using monthly calendars) and mobility-related disability (measured by an assessor masked to group allocation at baseline, 3 and 12 months). A fall was defined as an event in which the person unintentionally came to rest on the ground or other lower level, which was not as a result of violent blow, loss of consciousness or sudden onset of paralysis [14]. The performance-based mobility measure was the lower extremity component of the Short Physical Performance Battery [18] a composite score of three timed mobility measures, which has been found to predict adverse outcomes such as mortality and nursing home admission. The results of the trial indicated an improvement in mobility, measured using the Short Physical Performance Battery, in the intervention group compared to the control group (between-group difference in continuously scored version of Short Physical Performance Battery range 0–3 adjusted for baseline performance 0.13, 95 % CI 0.04 to 0.21, *p* = 0.004) but also a significant increase in falls in the intervention group compared to the control group (incidence rate ratio 1.43, 95 % CI 1.07 to 1.93, *p* = 0.017).

### Intervention and control groups

The program aimed to enhance mobility and prevent falls among older people after recent hospital stays. The intervention consisted of 10 home visits provided by an experienced physiotherapist over a twelve-month period. Exercises were based on the Weight-bearing Exercise for Better Balance (WEBB) program. This program involves simple exercises undertaken in weight-bearing positions that have been previously found to enhance physical functioning in older people [19, 20]. Participants were requested to undertake a 20–30 min program of exercises up to 6 times per week at home for twelve months. A full description of the exercise program is detailed in the protocol paper [15]. Participants in both groups received all usual care from local health and social support services as well as General Practitioners (GPs) and were provided with a fall prevention booklet. Control group participants did not receive any intervention as part of the study. Participants who commenced the program received an average of 9.2 (SD1.7) home visits. At the end of the 12 months, 61 % of the intervention group continued to exercise and, among the exercisers, the average number of exercise sessions completed was 4 per week [14].

### Data collection and outcome measures

Health-system and social support service contact, the focus of the present analyses, were secondary outcome measures in the trial. Participants were asked to record use of health and social support services on monthly calendars mailed to the research centre. Telephone contact was made with participants to obtain this information if calendars were not received. Demographic data were obtained at baseline, prior to randomisation, from medical record reviews and participant interviews.

Health service utilisation included residential care, hospital admissions, emergency department presentation, medical services and allied health services. Medical services incorporated General Practitioner and specialist services and community nursing intervention. Allied health included physiotherapy and occupational therapy services. Social support service utilisation data were derived as occasions of service of home care, the provision of housework, transport and shopping assistance and the number of meals provided.

Occasions of service delivery, considered as professional consultations or number of social support service visits were tabulated. Cost estimates were derived by multiplying the occasions of service delivery by the unit costs (valued in 2012 Australian dollars). Unit costs were obtained from the Medicare Benefits Schedule 2012 [21] or medical and allied health services, from Australian Refined Diagnosis Related Group cost weights [22] (AR-DRG version 5.2) for hospital admissions and emergency department presentations. The unit cost of emergency department presentation is based on an average estimate of $451 for all triaged categories, whereas hospital admission costs were specific to the diagnosis and the length of hospital stay of each patient. An average cost per hospital day was derived for this population group ($844) and was used where the information on “cause of hospital admission” was missing. An average cost per bed day for residential care was obtained from government sources and was estimated at $A94.79 per bed day [23]. The unit costs of social support services were obtained from a recent systematic review conducted in 2012 [24]. Health system contact was evaluated for all study participants, whereas residents of low-care aged facilities (hostels) were excluded from analysis of social support services, as these services were provided in-house by the aged care facility. The allocated per annum cost of a low-care residential facility was estimated at $A11,472.

### Statistical analyses

Resource utilisation was described using means, standard deviations, medians and interquartile ranges over the 12-month study period for the total sample. Rates of service use were compared between groups using negative binomial regression. Median costs in the two groups were compared using the Mann-Whitney *U* Test. Analyses were conducted using SPSS (version 21, IBM SPSS statistics, Armonk NY) and Stata (version 12, StataCorp LP, College Station, Texas) statistical computing packages. The main trial study was powered to detect an impact of the intervention on falls and therefore the between-group comparison of service use and costs is likely to be under-powered. This therefore is considered an exploratory analysis to assess the size and direction of any impact.

## Results

Participant characteristics are shown in Table 1. Forty-two percent of participants had originally been admitted to hospital due to a fall. Of these, 85 people were admitted with fractures and the most common fracture location was the hip or thigh (*n* = 50). For participants whose hospital admission was non-fall related, the most common reason for admission was a joint replacement procedure. Twenty participants resided in a hostel. The average length of the initial period of hospitalisation was 10 days.

**Table 1:** Participant characteristics at baseline (*n* = 340)

Characteristic	Control mean (SD) or n (%)	Intervention mean (SD) or n (%)	Total mean (SD) or n (%)
Female	128 (76 %)	123 (72 %)	251 (74 %)
Male	41 (24 %)	48 (38 %)	89 (26 %)
Age, years	81 (8)	82 (8)	81 (8)
Weight loss ≥ 5 % body weight	23 (14 %)	16 (9 %)	39 (12 %)
Co-morbidities, number	6.9 (2.7)	7.2 (2.9)	7.1 (2.8)
History of falls	52 (31 %)	48 (28 %)	100 (29 %)
Falls as the presenting problem to hospital	67 (40 %)	76 (44 %)	143 (42 %)
Length of original hospital stay^*^	9 (12)	11 (15)	10 (13)
Living in low-care residential facility	12 (7 %)	8 (5 %)	20 (6 %)
** MMSE score, mean (SD)/30	28.0 (1.8)	27.9 (2.0)	28.0 (1.9)

^*^Median and IQR shown due to skewed data

^**^MMSE: Minimental Status Examination Score

### Service utilisation

Of the 340 participants who were randomised, 337 provided service use and cost data, 18 had incomplete information in relation to the reason for hospital admission and the average cost of a hospital day was therefore used in cost-estimation. Twelve months of data were not available for 35 participants; nineteen participants died within the twelve-month study period, six withdrew and ten did not have complete health services data.

Table 2 shows the occasions of service delivery, number of users and the average per patient use for each resource used over the twelve-month study period.

**Table 2:** Occasions of service and number of users over the twelve-month study period

	Occasions of service	No of users	Percentage of the group utilising services	Average^a^ (SD)	Median^b^ (IQR)
Health and residential care services *n* = 337					
Hospital admissions	192	112	33 %	0.57 (21.63)	0.00 (0.50)
Residential high care	3	3	1 %	0.009 (0.25)	0.00 (0.00)
Residential low care	21^c^	21	6 %	0.06 (0.25)	0.00 (0.00)
ED presentations	39	33	10 %	0.12 (0.39)	0.00 (0.00)
GP	3,333	333	99 %	9.89 (6.49)	9.00 (3.50)
GP-home	256	55	16 %	0.76 (2.67)	0.00 (0.00)
Specialist	748	189	56 %	2.22 (3.72)	1.00 (5.00)
Nursing	1,263	68	20 %	3.75 (16.20)	0.00 (1.50)
Physiotherapy	975	83	25 %	2.89 (3.75)	0.00 (0.00)
Occupational therapy	59	22	7 %	0.18 (0.89)	0.00 (0.00)
Social support services in community-dwellers *n* = 319 (excludes participants living in low-care residential facility)					
Showering/dressing/personal care	4,980	58	18 %	15.61 (46)	0.00 (0.00)
Meals assistance	12,409	92	29 %	38.90 (88)	0.00 (21.00)
Domestic services	8,153	190	60 %	25.56 (49)	11 (16.00)
Transport	3,586	123	39 %	11.24 (29)	0.00 (5.00)
Shopping	2,206	71	22 %	6.92 (24)	0.00 (0.00)

^a^average occasions of service per participant ^b^median occasions of service per participant

^c^20 low care residents at baseline, 1 person admitted to a hostel during 12 month study-period

In total, 112 (33 %) participants were re-admitted to hospital during the twelve-month study period. The length of stay for each hospital admission ranged from 1 to 121 days. The average length of hospital stay (LOS) was 13 days. This is compared to the national average of a hospital admission in Australia of 3.12 days [22]. Thirty-four participants (10 %) were readmitted to hospital on more than one occasion. Most re-admissions were as a result of fall injuries (*n* = 36) but there were a range of other causes including infection, treatment of ulcers, investigation of gastrointestinal complaints and cardiovascular system complaints. Several had scheduled procedures including total hip or knee replacements, cataract surgery or other orthopaedic-type procedures. A small number of participants were admitted for residential care placement (*n* = 3).

The most widely used service was General Practitioner consultation in the consultation room, with almost all participants using this service. Overall, there was limited use of several services including nursing and allied health services, with the percentage of use in the sample ranging from 7 % for occupational therapy services to 25 % for physiotherapy. Use of social support services varied from 18 % for personal care to 60 % of the sample for domestic (home cleaning and gardening) activities.

### Cost estimates

Table 3 describes the average and the median cost for the range of services used over twelve-month study period. The estimated average cost expenditure for all services was $A10,091 per participant. Total costs were calculated at $A3,400,790 with most expenditure (56 %) attributed to hospital admission. The average cost per hospital admissions was $A16,846 compared to the national average of $A4,133 [22]. Expenditure on social support services accounted for almost 22 % of the total cost. The costs of allied health services contributed less than 2 % of the total cost.

**Table 3:** Average and median costs of services ($A2012)

Resource use	Unit costs	All participants *n* = 337		
		Average cost per patient (SD)	Median (IQR)	Total cost
Health and residential care services *n* = 337				
Hospital admission	DRG specific	$5,550 ($15,167)	$0.0 ($2,102)	$1,886,777
Residential high care	$94.79 bed day	$112 ($1,816)	$0.0 ($0.0)	$37,536
Residential low care	$11,472pa	$681 ($2,765)	($0.0) ($0.0)	$229,440
Medical services		$1,289 ($1,189)	$1,019 ($900)	$422,707
ED presentation	$451	$53 ($176)	$0.0 ($0.0)	$17,589
GP	$70.30	$720 ($457)	$633 ($492)	$234,310
GP-home	$81.85	$63 ($219)	$0.0 ($0.0)	$20,954
Specialist	$105.48	$243 ($393)	$105 ($316)	$78,899
Nursing	$56.18	$209 ($774)	$0.0 ($0.0)	$70,955
Allied health services		$222 ($502)	$0 ($64)	$62,040
Physiotherapy	$55.44 to $64.36	$173 ($208)	$0.0 ($64)	$58,500
Occupational therapy	$55.44 to $64.36	$10 ($74)	$0.0 ($0.0)	$3,540
Social support services *n* = 319, excludes participants living in low-care residential facility				
Social support services		$2,390 ($4,304)	$894 ($3,463)	$762,290
Showering/dressing	$36.40	$576 ($1,715)	$0.0 ($0.0)	$181,272
Meals	$11.10	$439 ($985)	$0.0 ($433)	$137,740
Domestic services	$39.07	$999 ($1,913)	$430 ($407)	$318,537
Transport	$12.39	$139 ($358)	$0.00 ($130)	$44,443
Shopping	$36.40	$252 ($878)	$0.00 ($0.00)	$80,298

### Group differences

There was no significant between group difference in the occasions of service for residential carer, hospital admission, allied health and social support services (Additional file 1: Table S1). There were also no between group differences for the majority of medical services including specialist and nursing services in the home or in consultation rooms but there was a significant between group difference for GP services provided in the home, with the intervention group recording a reduction in these services (IRR 0.23, 95 % CI -0.08 to 0.l54, *p* < 0.01). Significance testing did not indicate a between group difference in costs for any of the service categories (Additional file 1: Table S2). No clear trends in favour of either group were evident across the various types of services or costs.

## Discussion

The results indicate substantial use of health and support services in older people following hospital discharge, with an estimated average cost expenditure of $A10,091 per participant. The results confirm the significant risk of re-admission to hospital in older people with previous hospitalisation. The results also indicate widespread use of medical consultations in professional rooms (99 % of participants), comparatively little use of allied health services (32 %), greater use of domestic services (60 %) and less use of personal care services (18 %). There did not appear to be any consistent influence of the exercise intervention on resource use.

The most significant costs were associated with hospital admission, accounting for almost 56 % of the total expenditure. This is consistent with the findings of other studies [25, 26]. It should be highlighted that a small number of participants from the total group, around one-third, incurred the largest portion of the total cost expenditure. The average cost of hospital separation for this sub-group of $16,846 is consistent with that reported for fall-related hospital admission in the literature [27] and is four times the national average cost of a hospital separation [22]. The varied resource use of social support services with the greatest reliance on assistance with domestic services may reflect the reduced physical capacity of this older population group. There was little use of allied health service, which may have the potential to enhance physical capacity. Actual use of resources however may not be the sole indicator of need for these services, as there may be inequity in access to services, which is more dependent on resource availability and financial considerations.

The results also indicate that the exercise program did not seem to influence resource utilisation for the majority of services. One plausible explanation could be the older age of this group. Although the study included people aged 60 years and older, the average age of participants in this study was over 80 and as such, it may be that pre-hospitalisation use of medical, allied health and social support services was well-established and unlikely to alter in response to any provided intervention. The significantly lower use of home-based medical consultations in the intervention group may be due to home visits by the physiotherapist that were part of the study intervention. This suggests that a home visit by an allied health professional could reduce the perceived need for a home consultation by the general medical practitioner in this population.

The outcomes of the randomised controlled trial indicated an increase in falls but an improvement in mobility measures for the intervention group. It should be acknowledged however that a more intensive intervention might have led to different outcomes. Nevertheless the noted improvement in mobility was not reflected in a decrease in service use. Similarly, despite the increase in falls there was also no corresponding increase in service use or in the incidence of hospitalisation. This suggests that the falls did not have a significant cost impact on independence in the home environment resulting in the need for more services. There are several factors that may impact on the use of medical, allied health and social support services however, including personality type, culture and behavioural issues or financial affordability [4].

This study also provides an overview of the range of services used by older people and the associated costs of these services, assisting policy makers in planning for the health-care costs of the ageing population and providing information that allows consideration of various strategies for cost reduction. The widespread use of services by older people emphasises the need for good discharge planning and indicates that the continuum of care extends well beyond the period of hospitalisation. For the clinician this information is vital for the comprehensive assessment of patient needs with the literature findings demonstrating that well-coordinated care services post hospitalisation can prevent the progression of disability and improve functional outcomes [6].

Consideration of the extensive range of costs is also required in economic evaluation [28]. However, economic evaluations seeking to determine the cost-effectiveness of specific treatment intervention do not always consider the full range of allied health and social support services. The results of this study indicate that social support service costs constitute a substantial portion of total costs, and therefore should be taken into account when estimating the cost-effectiveness of interventions.

Limitations of this study include reliance on self-reported data for cost estimation and evaluation of costs from a trial population, which may not be representative of all older people discharged from hospital. The study considered costs from the perspective of the health-care service provider rather than the broader societal perspective. Appreciation of the costs incurred through the provision of informal care [29] and the associated lost opportunity costs would provide a better indication of the societal impact but was beyond the scope of this project. Information on service use prior to the original hospital admission was also lacking and would have been useful in evaluation of the impact of hospitalisation on resource utilisation. The study was also not designed to detect a between group difference in costs, it is likely that a significantly larger sample size would be required for this type of analysis given the variability in costs. The apparent substantial use of services in older people following hospital discharge is consistent with the previously noted high growth in expenditures for non-hospital [30] and hospital-related [31] services by older people in the later decades of life. Further analysis to determine the cost effectiveness of intervention with regards to the gains made in the mobility measures in this trial will provide valuable information for clinicians and policy-makers.

## Conclusion

There appears to be substantial use of services in older people following hospital discharge. These findings can be used for service planning and economic modelling. There did not appear to be any influence of a home-based exercise program on resource use or costs for most service categories. Further exploration of the extent to which post-hospital discharge costs are avoidable and modifiable by interventions is warranted.

## Availability of data and materials

The dataset analysed for this article is available upon request to the corresponding author.

## Additional file

Additional file 1: Table S1. Occasions of service and number of users over the twelve-month study period by group. Table S2. Average and median costs of services, for the exercise, control groups and the total cohort (base year 2012). (DOCX 25 kb)

## References

[1] World Health Organization. Aging and Life Course. Geneva Switzerland 13. Accessed 07 Nov 2013. Availale from http://www.who.int>ageing.

[2] Commonwealth of Australia. Economic Implications of an Ageing Australia. In: Department of Employment and Workplace Relations, editor. Canberra: Commonwealth of Australia; 2005.

[3] Pretty J (2011). Productivity commission draft report ‘Caring for Older Australians’: What could a new system look like?. Australas J Ageing.

[4] Australian Institute of Health and Welfare (2012). Australia’s health 2012. Australia’s health series no.13. Cat. no. AUS 156.

[5] Australian hospital statistics 2011–12, Australian Institute of Health and Welfare. Canberra, 2013.

[6] Gill TM, Allore HG, Holford TR, Guo Z (2004). Hospitalization, restricted activity, and the development of disability among older persons. JAMA.

[7] Mahoney J, Sager M, Dunham NC, Johnson J (1994). Risk of falls after hospital discharge. J Am Geriatr Soc.

[8] Mahoney JE, Palta M, Johnson J (2000). Temporal association between hospitalisation and rate of falls after discharge. Arch Intern Med.

[9] Sigurdsson E, Siggeirsdottir K, Jonsson H, Gudnason V, Matthiasson T, Jonson B (2008). Early discharge and home intervention reduces unit costs after total hip replacement: results of a cost analysis in a randomised study. Int J Health Care Finance Econ.

[10] McCallum J, Simons L, Simons J, Wilson J, Sadler P, Owen A (1996). Patterns and costs of post-acute care: a longitudinal study of people aged 60 and over in Dubbo. Aust NZ J Public Health.

[11] Liu CJ, Latham NK (2009). Progressive resistance strength training for improving physical function in older adults. Cochrane Database Syst Rev.

[12] Pahor M, Blair SN, Espeland M, Fielding R, Gill TM (2006). Effects of a physical activity intervention on measures of physical performance: Results of the lifestyle interventions and independence for Elders Pilot (LIFE-P) study. J Gerontol A Bio Sci Med Sci.

[13] Gillespie LD, Robertson CM, Gillespie WJ (2012). Interventions for preventing falls in older people living in the community. Cochrane Database Syst Rev.

[14] Sherrington CLS, Vogler CM, Close JCT, Howard K, Dean CM, Heller GZ, Clemson L, O’Rourke SD, Ramsay E, Barraclough E, Herbert RD, Cumming RG (2014). A post-hospital home exercise program improved mobility but increased falls in older people: a randomised controlled trial. PLoS One.

[15] Sherrington C, Lord SR, Vogler CM (2009). Minimising disability and falls in older people through a post-hospital exercise program: a protocol for a randomised controlled trial and economic evaluation. BMC Geriatr.

[16] Folstein MF, Folstein SE, McHugh PR (1975). “Mini-Mental state”: a practical method for grading the cognitive status of patients for the clinician. J Psychiatr Res.

[17] Tombaugh TN, McIntyre NJ (1992). The mini-mental state examination: a comprehensive review. J Am Geriatr Soc.

[18] Guralnik JM, Simonsick EM, Ferruci L, Glynn RJ, Berkman LF, Blazer DG, Scherr PA, Wallace RB (1994). A short physical performance battery assessing lower extremity fucntion: association with self-reported disability and prediciton of mortality and nursing home admission. J Gerontol.

[19] Sherrington C, Lord SR, Herbert RD (2004). A randomized controlled trial of weight-bearing versus non-weight-bearing exercise for improving physical ability after usual care for hip fracture. Arch Phys Med Rehabil.

[20] Olivetti L, Schurr K, Sherrington C, Wallbank G, Pamphlett P, Kwan MM-S, Herbert RD (2007). A novel weight-bearing strengthening program during rehabilitation of older people is feasible and improves standing up more than a nonweight-bearing strengthening program: a randomised trial. Aust J Physiother.

[21] Department of Health & Ageing. Medicare Benefits Schedule, 2013. Commonwealth of Australia. www.medicareaustralia.gov.au/provider/medicare/mbs.jsp. Accessed 07 Jul 2013.

[22] Commonwealth Department of Health and Ageing. National Hospital Cost Data Collection Cost Report Round 14 (2009–10). Canberra: 2011.

[23] Commonwealth of Australia. Department of Health and Ageing. Aged care subsidies and supplements new rates of payments from 20 September 2013. Canberra 2013. http://www.dss.gov.au>agedcaresubsidies. Accessed 12 Dec 2013.

[24] Farag I, Sherrington C, Ferreira M, Howard K (2013). A systematic review of the unit costs of allied health and community services used by older people in Australia. BMC Health Serv Res.

[25] Brainsky A, Glick H, Lydick E (1997). The economic cost of hip fractures in community-dwelling older adults: a prospective study. J Am Geriatr Soc.

[26] Claesson L, Gosman-Hedstrom G, Johannesson M, Fagerberg B, Blomstrand C (2000). Resource utilisation and costs of stroke unit care integrated in a care continuum: A 1-year controlled, prospective, randomised study in elderly patients: the Goteborg 70+ Stroke Study. Stroke.

[27] Watson W, Clapperton A, Mitchell R (2010). The incidence and cost of falls injury among older people in New South Wales 2006/2007.

[28] Drummond MF (2005). Methods for the economic evaluation of health care programmes.

[29] Whitlatch CJ, Feinberg LF (2006). Family and friends as respite providers. J Aging Soc Policy.

[30] Payne G, Laperte A, Deber R, Coyte PC (2007). Counting backwards to health care’s future: Using time to death modelling to identify changes in end-of-life morbidity and the impact of aging on health care expenditures. Milbank Q.

[31] Kardamanidis K, Lim K, Da Cunha C (2007). Hospital costs of older people in New South Wales in the last year of life. Med J Aust.

