# Supplementary material for: Health and social support services in older adults recently discharged from hospital: service utilisation and costs and exploration of the impact of a home-exercise intervention
Source: BMC Geriatr. 2016 Apr 18;16:82. doi: 10.1186/s12877-016-0254-x (PMC4835837; doi:10.1186/s12877-016-0254-x)
Supplement: Additional file 1: Table S1. — Occasions of service and number of users over the twelve-month study period by group. Table S2. Average and median costs of services, for the exercise, control groups and the total cohort (base year 2012). (DOCX 25 kb) [file 12877_2016_254_MOESM1_ESM.docx]

Additional file 1

Table S1. Occasions of service and number of users over the twelve-month study period by group

| **Resource** | **All participants** | **Control**  **n = 167** | | | | **Intervention**  **n = 170** | | | | **Difference between groups** |
| --- | --- | --- | --- | --- | --- | --- | --- | --- | --- | --- |
|  | **Occasions of service** | **Occasions of service** | **No of users** | **Average** †  **(SD)** | **Median** ‡  **(IQR)** | **Occasions of service** | **No of users** | **Average**†  **(SD)** | **Median** ‡ **(IQR)** | **IRR between group difference**  **(CI; p-value)** |
| **Health and residential care services n=337** | | | | | | | | | | |
| Hospital admissions | 192 | 82^a^ | 48 | 0.49 (21.63) | 0.00 (1.00) | 110^b^ | 64 | 0.64 (13.57) | 0.00 (1.00) | 1.13 (0.57 to 2.24; 0.73) |
| Residential high care | 3 | 2^d^ | 2 | 0.01 (0.25) | 0.00 (0.00) | 1^d^ | 1 | 0.001 (0.25) | 0.00 (0.00) | § |
| Residential low care | 21 | 1 | 12^c^ | 0.07 (0.25) | 0.00 (0.00) | 0 | 8^c^ | 0.05 (0.25) | 0.00 (0.00) | § |
| ED presentations | 39 | 21 | 16 | 0.12 (0.43) | 0.00 (1.00) | 18 | 17 | 0.11(0.35) | 0.00 (0.50) | 0.89 (0.44 to 1.81; 0.74) |
| GP | 3,333 | 1,616 | 165 | 9.56 (6.61) | 11.00 (6.50) | 1,717 | 168 | 10.04 (6.40) | 11.00 (5.50) | 1.04 (0.92 to 1.18; 0.50) |
| GP-home | 256 | 210 | 33 | 1.24 (3.62) | 0.00 (0.00) | 46 | 22 | 0.29 (0.90) | 0.00 (0.00) | 0.22 (0.10 to 0.48;0.00) |
| Specialist | 748 | 380 | 93 | 2.40 (3.92) | 2.00 (5.00) | 368 | 96 | 2.20 (3.54) | 2.00 (4.00) | 0.95 (0.67 to 1.33; 0.74) |
| Nursing | 1,263 | 833 | 33 | 4.93 (16.20) | 0.00 (1.50) | 430 | 35 | 2.52 (10.86) | 0.00 (4.00) | 0.51 (0.22 to 1.21; 0.13) |
| Physiotherapy | 975 | 509 | 47 | 3.02 (4.17) | 0.00 (2.00) | 466 | 36 | 2.73 (3.29) | 0.00 (1.00) | 0.79 (0.37 to 1.68; 0.53) |
| Occupational therapy | 59 | 24 | 10 | 0.14 (0.89) | 0.00 (0.00) | 35 | 12 | 0.21 (1.37) | 0.00 (0.00) | 1.45 (0.47 to 4.80; 0.55) |
| **Social support services in community-dwellers n = 319 (excludes participants living in low-care residential facility)** | | | | | | | | | | |
| Showering/dressing/  home care | 4,980 | 2,463 | 31 | 15.89 (46) | 0.00 (0.00) | 2,517 | 27 | 15.44 (49) | 0.00 (0.00) | 0.93 (0.27 to 3.22; 0.91) |
| Meals assistance | 12,409 | 5,886 | 47 | 37.97 (84) | 0.00 (60.00) | 6,523 | 45 | 40.27 (93) | 0.00 (36.00) | 1.04 (0.37 to 2.91; 0.94) |
| Domestic services | 8,153 | 3,177 | 93 | 20.63 (35) | 7 (28.00) | 4,976 | 97 | 30.52 (59) | 12.00(40.25) | 1.39 (0.86 to 2.26; 0.18) |
| Transport | 3,586 | 1,486 | 63 | 9.65 (26) | 0.00 (11) | 2,101 | 60 | 12.89 (32) | 0.00 (9.75) | 1.29 (0.66 to 2.54; 0.46) |
| Shopping | 2,206 | 780 | 34 | 5.03 (17) | 0.00 (0.00) | 1,426 | 37 | 8.80 (30) | 0.00 (00.00) | 1.57 (0.57 to 4.30; 0.38) |

† average occasions of service per participant ‡ median occasions of service per participant  § numbers considered too small to run analyses ^a^ 676 days in hospital, median (IQR) length of stay 7 (14) days, ^b^ 789 days in hospital, median (IQR) length of stay 7 (17) days ^c^ low care residents at baseline , ^d^ admitted during 12 month study-period

Table S2: Average and median costs of services, for the exercise, control groups and the total cohort (base year 2012)

| **Resource use** | **Unit costs** | **All participants**  **n=337** | | **Control**  **n=167** | | | **Intervention**  **n=170** | | | **Difference between groups** |
| --- | --- | --- | --- | --- | --- | --- | --- | --- | --- | --- |
|  |  | **Average cost per patient**  **(SD)** | **Median (IQR)** | **Average cost per patient**  **(SD)** | **Median (IQR)** | **Total cost** | **Average cost per patient**  **(SD)** | **Median**  **IQR** | **Total**  **cost** | **Median difference**  **(CI; p-value)** |
| **Health and residential care services n=337** | | | | | | | | | | |
| Hospital admission | DRG specific | $5,550  ($15,167) | $0.0 ($2,102) | $4922  ($15,579) | $0.0  ($1,172) | $826,748 | $6,199  ($14809) | $0.0  ($2483) | $1,060,029 | $0 ($0 to $0; 0.32) |
| Residential high care | $94.79 bed day | $112  ($1,816) | $0.0  ($0.0) | $77  ($895) | $0.0  ($0.0) | $12,891 | $145  ($1,885) | $0.0  ($0.0) | $24,645 | $0 ($0 to $0; 0.32) |
| Residential low care | $11,472pa | $681  ($2,765) | ($0.0) ($0.0) | $824  ($2,584) | ($0.0) ($0.0) | $137,664 | $540  ($2,939) | ($0.0) ($0.0) | $91,776 | $0 ($0 to $0; 0.25) |
| **Medical services** |  | **$1,289**  **($1,189)** | **$1,019**  **($900)** | **$1,387**  **($1,326)** | **$1,055**  **($922)** | **$227,145** | **$1,192**  **($1032)** | **$984**  **($902)** | **$195,562** | **$70 (-$70 to $211; 0.29)** |
| ED presentation | $451 | $53  ($176) | $0.0 ($0.0) | $56  ($192) | $0.0  ($0.0) | $9,471 | $50  ($158) | $0.0  ($0.0) | $8,118 |  |
| GP | $70.30 | $720  ($457) | $633 ($492) | $706  ($465) | $633  ($422) | $113,605 | $733  ($450) | $703  ($492) | $120,705 |  |
| GP-home | $81.85 | $63  ($219) | $0.0 ($0.0) | $103  ($296) | $0.0  ($0.0) | $17,189 | $35  ($74) | $0.0  ($0.0) | $3,765 |  |
| Specialist | $105.48 | $243  ($393) | $105 ($316) | $251  ($413) | $105  ($316) | $40,082 | $236  ($373) | $105  ($316) | $38,817 |  |
| Nursing | $56.18 | $209  ($774) | $0.0 ($0.0) | $277  ($910) | $0.0  ($0.0) | $46,798 | $141  ($610) | $0.0  ($0.0) | $24,157 |  |
| **Allied health services** |  | **$222**  **($502)** | **$0**  **($64)** | **$207**  **($434)** | **$0**  **($215)** | **$31,980** | **$238**  **($561)** | **$0**  **($257)** | **$30,060** | **$0 ($0 to $0; 0.61)** |
| Physiotherapy | $55.44 to $64.36 | $173  ($208) | $0.0  ($64) | $181  ($231) | $0.0  ($64) | $30,540 | $164  ($182) | $0.0  ($64) | $27,960 |  |
| Occupational therapy | $55.44 to $64.36 | $10  ($74) | $0.0 ($0.0) | $9  ($57) | $0.0  ($0.0) | $1,440 | $12  ($88) | $0.0  ($0.0) | $2,100 |  |

| **Social support services n = 319, excludes participants living in low-care residential facility** |
| --- |

| **Social support services** |  | **$2,390**  **($4,304)** | **$894**  **($3,463)** | **$2,103**  **($3,205)** | **$843**  **($3,621)** | **$325,917** | **$2,677**  **($5,138)** | **$938**  **($3,081)** | **$436,373** | **$0 (-$148 to $12; 0.81)** |
| --- | --- | --- | --- | --- | --- | --- | --- | --- | --- | --- |
| Showering/dressing | $36.40 | $576  ($1,715) | $0.0 ($0.0) | $578  ($1,666) | $0.0  ($0.0) | $89,653 | $562  ($1,766) | $0.0  ($0.0) | $91,619 |  |
| Meals | $11.10 | $439  ($985) | $0.0 ($433) | $421  ($932) | $0.0  ($666) | $65,335 | $444  ($1,027) | $0.0  ($400) | $72,405 |  |
| Domestic services | $39.07 | $999  ($1,913) | $430 ($407) | $801  ($1,347) | $234  ($1,094) | $124,125 | $1,193  ($2,319) | $469  ($1,563) | $194,412 |  |
| Transport | $12.39 | $139  ($358) | $0.00 ($130) | $119  ($315) | $0.0  ($135) | $18,412 | $160  ($395) | $0.0  ($111) | $26,031 |  |
| Shopping | $36.40 | $252  ($878) | $0.00 ($0.00) | $183  ($607) | $0.0  ($0.0) | $28,392 | $318  ($1,073) | $0.0  ($0.0) | $51,906 |  |
